# Processing of Abstract Rule Violations in Audition

**DOI:** 10.1371/journal.pone.0001131

**Published:** 2007-11-07

**Authors:** Erich Schröger, Alexandra Bendixen, Nelson J. Trujillo-Barreto, Urte Roeber

**Affiliations:** 1 Institute of Psychology I, University of Leipzig, Leipzig, Germany; 2 Cuban Neuroscience Center, Havana, Cuba; University of Minnesota, United States of America

## Abstract

The ability to encode rules and to detect rule-violating events outside the focus of attention is vital for adaptive behavior. Our brain recordings reveal that violations of abstract auditory rules are processed even when the sounds are unattended. When subjects performed a task related to the sounds but not to the rule, rule violations impaired task performance and activated a network involving supratemporal, parietal and frontal areas although none of the subjects acquired explicit knowledge of the rule or became aware of rule violations. When subjects tried to behaviorally detect rule violations, the brain's automatic violation detection facilitated intentional detection. This shows the brain's capacity for abstraction – an important cognitive function necessary to model the world. Our study provides the first evidence for the task-independence (i.e. automaticity) of this ability to encode abstract rules and for its immediate consequences for subsequent mental processes.

## Introduction

The capacity to encode and apply abstract rules is beneficial for adaptive behavior in our complex environment, in which often relations between stimuli (rather than stimuli per se) are constant. Most of our knowledge about extraction and utilization of rules, however, is based on the simulation of simple environments in which a very limited stimulus set is used, and in which rules are characterized by stimulus repetitions (concrete rule) rather than constant relations between stimuli (abstract rule). While the extraction of concrete rules relies on the constancy of specific feature values of the respective stimuli, extraction and application of abstract rules also works with stimuli the organism has never encountered before. By exploiting constant relationships between features, the stimulus features per se can vary, but rules related to the relationships can still be detected. The encoding of rules is well investigated when rules are task-relevant[1], [2] or when they apply to motor behavior[3]. In contrast, the unintentional encoding and application of rules that are not relevant for the current task received only little attention[4], and even less so with abstract rules[5], [6]. It is this ability which considerably increases our possibilities to model the world and enables adaptive behavior[7]. In the present study, we investigated the brain's ability to automatically encode abstract rules and to register events violating them, as well as its consequences for other mental processes, namely the interference with the processing of task-relevant information and the facilitation of behavioral detection of rule violations.

Evidence for the existence of unintentional encoding of auditory rules has been provided by several passive oddball studies using the Mismatch Negativity (MMN) brain wave of the event-related potential (ERP)[5], [8]–[10]. However, experimental research is still sparse and mainly focused on the type of rules that can be encoded automatically rather than on the consequences of abstract rule encoding. The present study investigates the chronometric dynamics and the neuroanatomical sources of the unintentional detection of abstract rule violations, and, most importantly, its consequences on other cognitive processes depending on task demands. By manipulating attentional allocation to and task relevance of the rules, we determined the degree of automaticity of the processes underlying the detection of abstract rule violations. Scalp current density analyses (SCD)[11] and primary current density (PCD) analyses with variable resolution electromagnetic tomography (VARETA)[12]–[14] were applied to reveal the cortical areas involved.

High-density EEG recordings were taken from subjects presented with sound sequences obeying an abstract rule, including rare violations of this rule in different experimental conditions. In order to minimize influences of cognitive control on our results, rules were constructed in a way that they could hardly be consciously noticed by untrained participants.

In one condition, in which subjects ignored the sounds, it was tested whether establishment and application of abstract auditory rules as indexed by the MMN occurs unintentionally. In another condition, subjects performed a task related to the sounds but not related to the rule. With concrete rules it has been shown that rule violations not only elicit MMN but also P3a, an ERP indicator of involuntary attention switching, and cause behavioral impairment in the primary task (prolongation of reaction times and decrease of hit rates)[15]. It is of interest whether violations of abstract rules can also interfere with task-related processing and whether the network involved includes supratemporal, parietal and frontal areas which are known to be involved in auditory distraction[16], [17]. In a third condition, subjects not only attended to the sounds (as in the Distraction condition), but rule violations had to be detected, that is, they became task-relevant. As the rule was constructed to be rather difficult for the subjects to encode and apply, trials could be analyzed separately for behaviorally detected and undetected rule violations. Provided that the intentional detection of rule violations is governed by sensory-memory representations indicated by the MMN[18], the brain waves are expected to differ between detected and undetected violations.

## Results

### Ignore Condition

**Figure 1:**
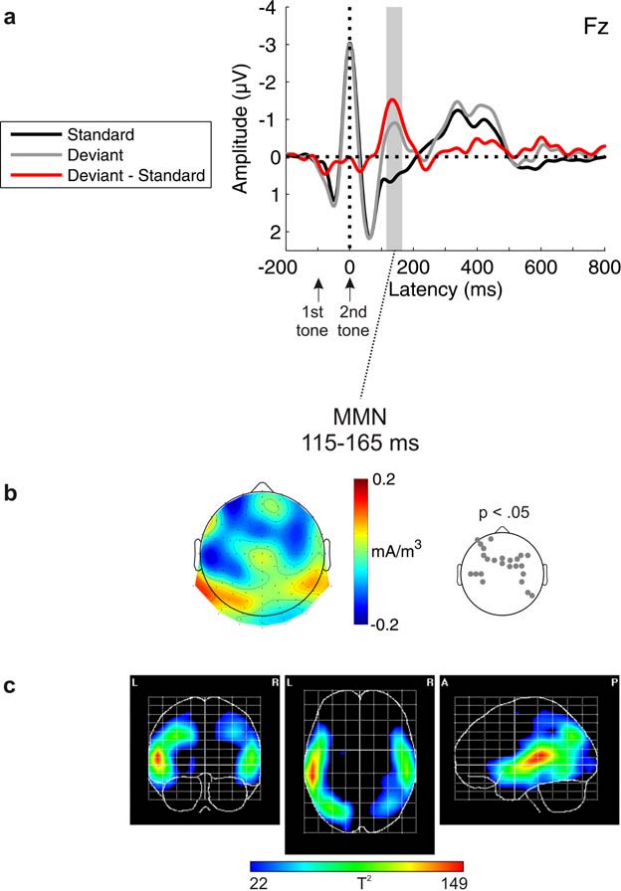
Ignore condition for abstract rules. a) Grand-average ERPs elicited by standards (black) and deviants (gray), and deviant minus standard difference wave (red). b) Topographic distribution of the MMN component (scalp current density, SCD). Electrode positions with SCD values significantly deviating from zero are indicated in the p-value map. c) Tomographic distribution of the MMN component (primary current density, PCD). The hotter colors correspond to higher probability values (one-way ANOVA; thresholded to p<.0001).

Violations of the frequency relation between the two tones in a pair elicited the MMN [t(11) = −3.830, p = .001] revealing a typical time-course and topographical distribution (i.e., fronto-central negativity and polarity inversion at postero-temporal leads; Fig. 1a). The respective SCD (Fig. 1b) points at generators in supratemporal areas. This is supported by the PCD analysis (Fig. 1c) yielding intracerebral sources with maxima in the superior temporal gyri of both hemispheres extending into the inferior frontal gyri. Additional activations were found with local maxima in parieto-occipital areas (superior parietal lobuli, angular gyri, middle occipital gyri).

### Distraction Condition

#### Behavioral results

The duration discrimination task was resolved quickly (mean reaction time: 449 ms) and accurately (hit rate: 97%). RTs were prolonged [t(11) = −3.689, p = .004], and hit rates were reduced [t(11) = 2.442, p = .033] in Deviant as compared to Standard trials. In other words, subjects' performance was impaired by task-irrelevant deviations. Importantly, subjects neither acquired explicit knowledge of the rule nor became aware of the presence of rule violations.

#### Electrophysiological results

**Figure 2:**
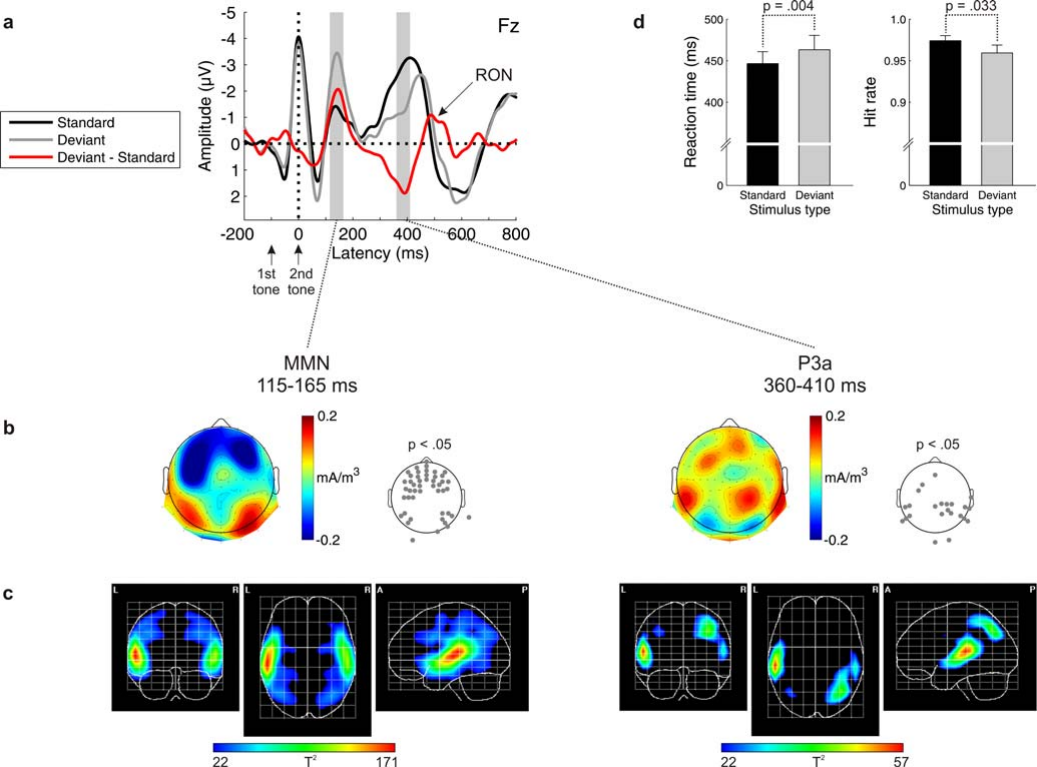
Distraction condition for abstract rules. a) Grand-average ERPs elicited by standards (black) and deviants (gray), and deviant minus standard difference wave (red). b) Topographic distributions of the MMN and P3a components (scalp current density, SCD). Electrode positions with SCD values significantly deviating from zero are indicated in the p-value maps. c) Tomographic distributions of the MMN and P3a components (primary current density, PCD). The hotter colors correspond to higher probability values (one-way ANOVA; thresholded to p<.0001). d) Behavioral data for standard and deviant tone pairs.

MMN was obtained [t(11) = −3.785, p = .002] (Fig. 2). Its amplitude, time-course, and distribution was similar to the one obtained in the Ignore condition. SCD and PCD analyses confirmed generators in auditory areas (superior temporal gyri). The PCD analysis showed extended activations in frontal (middle frontal and inferior frontal gyri) and parieto-occipital areas (superior parietal lobuli, angular gyri, middle occipital gyri). Subsequent to MMN, P3a was elicited [t(11) = 4.027, p = .001]. SCD and PCD analyses suggest contributions from superior temporal gyri, right middle frontal gyrus, and right parieto-occipital areas (superior parietal lobuli, angular gyrus, middle occipital gyrus).

### Detection Condition

**Figure 3:**
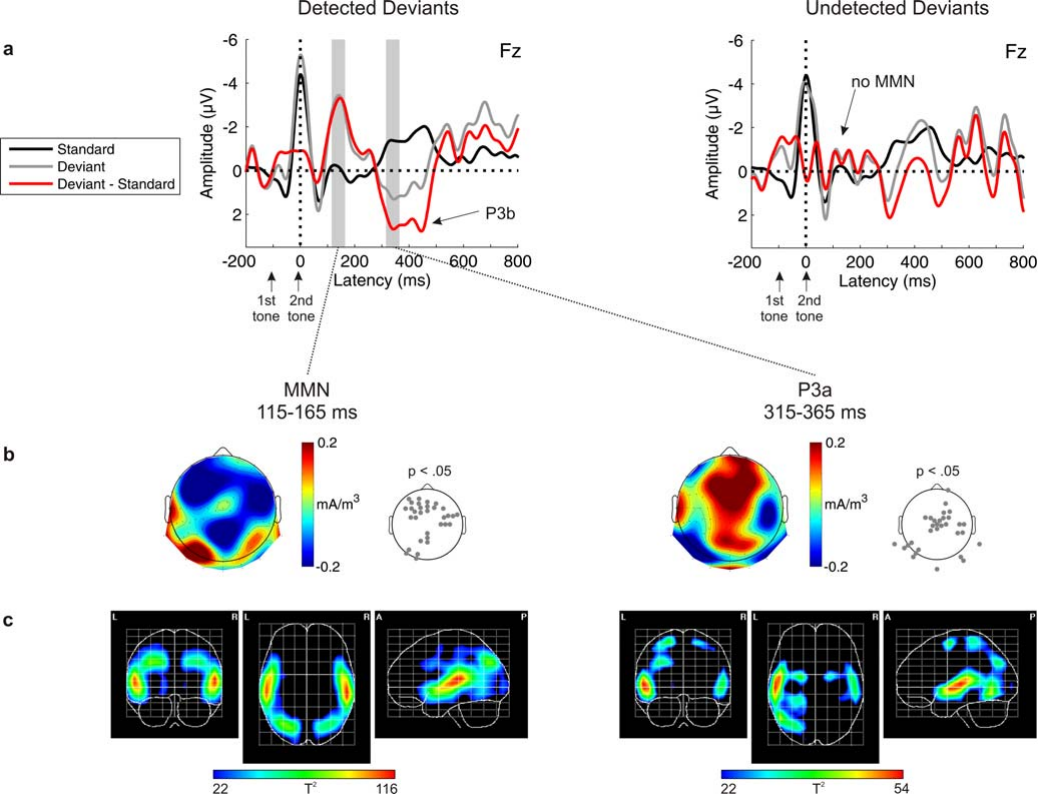
Detection condition for abstract rules. a) Grand-average ERPs elicited by standards (black) and deviants (gray), and deviant minus standard difference wave (red) according to detection performance (detected deviants, left; undetected deviants, right). Note that the ERPs for undetected deviants are noisier because for some subjects, only few trials with undetected deviants were observed. However, exclusion of those subjects does not change the results, i.e. MMN is still not present for undetected deviants. b) Topographic distributions of the MMN and P3a components for detected deviants (scalp current density, SCD). Electrode positions with SCD values significantly deviating from zero are indicated in the p-value maps. c) Tomographic distributions of the MMN and P3a components for detected deviants (primary current density, PCD). The hotter colors correspond to higher probability values (one-way ANOVA; thresholded to p<.0001).

After explicit instruction about the rule and behavioral training to detect rule violations, subjects performed the Detection condition. The average detection rate of rule violations was 72%. ERPs for detected rule violations show MMN [t(11) = −5.930, p = .000] and P3 [t(11) = 2.465, p = .016], while ERPs elicited by undetected rule violations are rather similar to the ERPs elicited by the standard tone pairs, i.e. no MMN was elicited [t(11) = −0.677, p = .256] (Fig. 3). Similar to the Ignore and Distraction conditions, sources for MMN were again found in superior temporal, frontal, and parieto-occipital areas. The P3 consisted of two separate peaks, suggesting two separate processes contributing to the deviance-related effect. For the early part of P3, the PCD analysis revealed activations in the superior temporal gyri, superior frontal gyri, left postcentral gyri, left superior parietal lobe, left angular gyrus, and left occipitotemporal gyrus. It seems likely that this early peak is dominated by the P3a, while the later peak mainly consists of P3b usually elicited by (rare) target stimuli[19]. However, as both components may overlap in time, both may contribute to the deviance-related positivity.

## Discussion

In this work, we studied the unintentional encoding of the frequency relation between two tones in a tone pair and the unintentional detection of deviations from that relation as well as its functional role for other cognitive processes, namely the involuntary switching of attention towards events violating the regulation and the intentional detection of such events.

### Unintentional detection of abstract rule violations

Evidence for the brain's capacity to automatically detect violations of abstract rules has been reported previously[5], [6], [8], [10], [20]. However, most reports published so far have used a rather limited set of stimuli obeying the rule (7 to 20). Thus, it cannot completely be excluded that previous results are based on exemplar learning rather than on abstract rule establishment. Here, a larger set of stimuli was used (60) in a dynamic experimental setting in which sounds not only varied in frequency, but also in duration. Yet, occasional violations of the frequency relation resulted in the elicitation of MMN in subjects who were engaged in watching a subtitled video. This suggests that a constant relation between the frequencies of successive sounds within a tone pair is encoded by the auditory system.

Interestingly, the time-course of the MMN is rather similar to the one obtained when a concrete rule is defined on the frequency dimension (see Fig. S1, S2 and S3 of the Supplementary Material for the MMN elicited by the violation of a concrete rule within the same paradigm and subjects). This argues for a very efficient way of encoding frequency relations. One possibility how this can be achieved has been raised by Ulanovsky[21], who argued that stimulus-specific adaptation (SSA) mechanisms studied in anesthetized cats in single auditory cortex neurons could explain MMN to abstract changes measured in humans. In fact, SSA has been proposed to be a single neuron correlate of the MMN[22]. In principle, this seems possible as there exist neurons selectively responding to either ascending or descending frequency[23]. Moreover, the main source contribution to the present MMN has been localized in primary auditory cortex, where SSA occurs. Alternatively, mere cognitive accounts of the mechanisms underlying the present MMN also seem reasonable. MMN can be elicited by violations of concrete rules defined on the frequency or location dimensions when neural refractoriness is controlled for[24]. Functional magnetic resonance imaging (fMRI)[25] and magneto-encephalographic (MEG)[26] research controlling for refractoriness revealed that MMN for violations of concrete rules is also localized in auditory cortex.

Significant activations extended to inferior frontal and parieto-occipital areas. In a previous MEG study, sources of abstract rule MMN were confined to auditory cortex[27]. However, as MEG is mainly sensitive to tangentially oriented generators[28], frontal and parieto-occipital contributions to abstract rule MMN could hardly be detected with MEG[29]. However, frontal (e.g. frontal operculum and inferior frontal gyrus) and sometimes parietal (e.g. inferior and posterior parietal cortical areas) contributions to MMN elicited by concrete rule violations have been reported in several EEG[30], [31], fMRI[32]–[34], event-related optical imaging[35], PET[36], and patient[37], [38] studies. Thus, MMN for abstract rule violations seems to involve a similar network as MMN for concrete rule violations.

### Involuntary attention switch triggered by abstract rule violations

When subjects had to perform a two-alternative forced choice duration discrimination task, performance was modulated by the type of the frequency relation between stimuli. Reaction times were prolonged and hit rates decreased in trials where the rule was violated. This deterioration in task performance due to task-irrelevant rule violations is consistent with results from auditory distraction studies using concrete rules[17], [39], [40]. It shows that violations of abstract rules may affect the processing of task-relevant information. This result can best be explained within the context of involuntary attention. The observation that MMN is followed by P3a in the Distraction condition supports this hypothesis[4]. Mainly auditory but also frontal generators of MMN were found in the Distraction condition. The frontal contribution to MMN has been proposed to reflect an attention trigger signal which may initiate a subsequent attention switch[29], [41]. The present P3a had generators in auditory cortex and in middle frontal gyrus (MFg) with a right-hemispheric lateralization. MFg has been proposed to represent part of a ventral right-frontoparietal network engaged in exogenous orienting[16]. This network may serve as an alerting system detecting unexpected, behaviorally relevant stimuli in the environment. A recent fMRI study using a similar distraction paradigm with concrete (instead of abstract) rule violations yielded deviance-related activations in inferior frontal gyrus (IFG), medial frontal cortex, intraparietal sulcus (IPS), surpramarginal gyrus, and the temporo-parietal junction (TPJ)[17].

As intended, our subjects did not become aware of the abstract rule or of the occurrence of rule violations. It should be noted that techniques such as the generation of legal sequences often applied in implicit learning paradigms would have been too difficult in the present paradigm and could thus not be applied. Yet, even after careful interview, our subjects did not show any sign of explicit knowledge about the rule. The presence of MMN, P3a, and behavioral impairment in task performance suggests that deviations that are not noticed by the subjects can still interfere with task-related processes, activating a similar network as easily detectable concrete rule violations[17]. In this sense, effects of involuntary attentional orienting do not necessarily involve awareness of the presence of a distractor. If subjects know about the rules as they usually do in oddball studies, it cannot be excluded that rule encoding and application (indicated by the deviance-related effects) is in fact due to subjects' cognitive top-down control. Importantly, for the present Distraction condition, the absence of explicit knowledge about the rule and rule violations implies that the deviance-related processing is bottom-up driven rather than top-down controlled in nature.

### Intentional detection of rule violations

When subjects were asked to respond to rule violations, they managed to detect 72% of the tones violating the rule. The separate analysis of trials with detected and undetected rule violations revealed that MMN and P3a were confined to trials in which the rule violations were detected. This is consistent with the hypothesis that the behavioral deviance detection is governed by the processes underlying MMN generation[18], [20], which received various support from combined ERP and behavioral studies[42], [43]. However, the converse conclusion that every rule violation being registered unintentionally can also be detected behaviorally cannot be drawn, as it has been shown that MMN can be present while the violation is unnoticed by the subject[9], [44].

One may ask how it comes that MMN is elicited in some trials while it is not in others. It may either be the case that a particular violation is missed although the rule is represented, or that a sound being adequately encoded is not identified as a rule violation because the rule is currently not represented. Indeed, the dynamic stimulation of the present experimental protocol may result in varying perceptual organization/distinctness of the sounds depending on their local context, which could prevent that a sound is evaluated as violating an existing rule. On the other hand, the dynamic stimulation may also result in occasional cessation of the rule. Search for factors influencing the detection performance supported, if any, the first rather than the second alternative (see Fig. S4 of the Supplementary Material showing that violations were more likely to be detected when they occurred in a tone pair with the second tone being long or when the frequency separation to the previous tone pair was large). It seems likely that the MMN system may also have missed some rule violations in the Ignore and Distraction conditions.

### Conclusion

The initial brain response to sounds violating an abstract rule reveals that our brain encodes and applies such rules. The finding that this happens even when subjects do not attend the sounds supports the hypothesis that abstract rule encoding occurs unintentionally[5], [6], [8]–[10]. The temporal and structural characteristics of the relevant brain response (MMN) were virtually identical in all conditions, that is, when subjects were ignoring the sounds, when they attended the sounds but rules were task-irrelevant, and when they attended the sounds and rules were task-relevant. This shows that the processes accomplishing rule encoding and application are largely independent of the task, which is an important criterion for defining a mental process as automatic[45].

With this ability to encode and apply abstract rules, the brain can derive predictions about forthcoming events, even if it has not encountered these events before. We showed for the first time that this ability has consequences for adaptive behavior: First, task-irrelevant rule violations impaired behavioral performance in the primary task and activated a network previously found to be engaged in involuntary attention[16], [17]. Second, intentional detection of violations is (at least partly) based on the outcome of the unintentional mechanism. Thus, modelling the world by representing the rules inherent to relations between stimuli indeed aids the gathering of information required for adaptive behavior.

## Materials and Methods

### Participants

Twelve normal-hearing healthy subjects (four male, one left-handed, mean age 24 years) participated in the experiment for either course credit or payment (6 € per hour). The experiment was undertaken with the understanding and written consent of each subject. The experimental protocol conformed to the Declaration of Helsinki and the ethics guidelines of the German Association of Psychology (ethics board of the Deutsche Gesellschaft für Psychologie, DGPs: http://www.dgps.de/dgps/aufgaben/ethikrl2004.pdf) and did thus not require any additional ethics approval.

### Apparatus and procedure

Identical stimulation was presented in a passive and an active session, both comprising one part in which the sound sequences included an abstract rule and another part in which they comprised a concrete rule. The purpose of the concrete rule was to assess the presence of orderly deviance-related ERP (MMN, P3a) and behavioral effects (increase in RT and error rate in rule-violating trials relative to rule-conforming trials) with the present experimental protocol. Results and Discussion for the concrete rule are presented as Supplementary Material (Fig. S1, S2 and S3). Order of sessions and of rule types within each session was counterbalanced across subjects.

**Figure 4:**
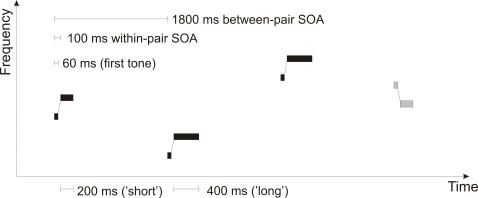
Stimulation for an abstract rule sequence. Tone pairs vary in their absolute frequencies. Standard tone pairs (black) are characterized by an ascending frequency relation, whereas deviant tone pairs (gray) are descending. Duration of the second tone in the pair varies randomly and independently of the frequency relations.

Pairs of sinusoidal tones (100 ms within-pair SOA, 1800 ms between-pair SOA) were presented via headphones with an intensity of ca. 70 dB SPL. Duration was 60 ms for the first tone and either 200 or 400 ms with equal probability for the second tone (both tones including 10 ms rise and 10 ms fall times). In concrete rule sequences, frequency of the first tone was 900 Hz; in abstract rule sequences, it was chosen randomly from 10-Hz steps in the interval of 600 to 1200 Hz (Fig. 4). For both rule types, frequency of the second tone was 26% higher than that of the first tone in 87.5% of the pairs (standards, “rising”) and 26% lower in the remaining 12.5% of the pairs (deviants, “falling”). Stimulus percentages were reversed for half of the subjects; results from subjects with “rising” and “falling” rules were later collapsed in averaging. Stimulus type (standard/deviant) and duration (short/long) were counterbalanced within subjects. Sound sequences were randomized individually for each subject.

In the Passive session, subjects watched a soundless, subtitled video and were instructed to ignore the stimuli (Ignore condition). In the Active session, they completed different tasks in different blocks, the frequency relation within the tone pair (i.e. the rule) either being task-irrelevant (Distraction condition) or relevant (Detection condition). In the Distraction condition, subjects performed a two-alternative forced-choice duration discrimination decision, judging the second tone of each pair as being short or long by pressing a button with the left or right index finger. At the end of the Distraction condition, subjects were interviewed in a standardized way in order to determine to what extent they acquired explicit knowledge about the (task-irrelevant) rule or its violations. In the Detection condition, which was always administered after the Distraction condition, subjects were informed about the rule (rising/falling) and were instructed to detect deviants and to indicate them by button presses. For both conditions, button-response assignment was counterbalanced across subjects.

Each block consisted of 160 tone pairs. Eight blocks per type of rule (Concrete/Abstract) were administered in the Passive session (80 minutes total duration). The Active session (60 minutes) comprised four blocks per type of rule for the Distraction condition and two blocks per type of rule for the Detection condition. The different number of blocks per condition was chosen because different signal-to-noise ratios were expected [46]. Depending on the subject's performance, duration discrimination was practiced before the experimental blocks of the Active session.

### Behavioral data

In the Active session, subjects' responses were recorded, and reaction times (RTs) were measured relative to the onset of the second tone for the Detection condition and relative to the onset of the duration difference (i.e. 200 ms after the onset of the second tone) for the Distraction condition.

### Electrophysiological data

Using a BIOSEMI Active-Two amplifier system, electroencephalographic (EEG) activity was continuously recorded from 128 standard locations according to the ABC electrode system where electrode positions are radially equidistant from CZ (http://www.biosemi.com/headcap.htm), and from the left and right mastoids. Electrodes were mounted in a nylon cap. Eye movements were monitored by bipolar horizontal and vertical EOG derivations. EEG and EOG recordings were sampled at 512 Hz. Offline, EEG activity was re-referenced to the activity recorded at the tip of the nose, and EEG and EOG activity was filtered (1.0 Hz high-pass, 20 Hz low-pass).

ERPs were obtained time-locked to the onset of the second tone within a pair by averaging epochs of 1000 ms duration (including a 100-ms baseline before the onset of the first tone) for each trial. Records were sorted as a function of the factors condition (Ignore/Distraction/Detection), type of rule (Concrete/Abstract), and stimulus type (Standard [confirming the rule]/Deviant [violating the rule]). For an additional analysis of the Detection condition, deviant ERPs were further subdivided according to behavioral performance (Detected/Undetected). Standards following a deviant were excluded from all analyses. Difference waves were formed by subtracting the ERPs elicited by standards from those elicited by deviants.

MMN and P3a amplitudes were measured from the individual difference waves as the mean signal amplitude in 50-ms intervals around the latency of the grand-average ERP component peaks. Presence of the MMN and P3a components was verified by testing their mean amplitudes against zero via one-sample, one-tailed Student's *t*-tests at a significance level of .05. Using a two-dimensional spherical spline interpolation, scalp potential maps were generated in order to analyze the spatiotemporal structure with a higher spatial resolution. Scalp current density (SCD) distributions were estimated from the surface laplacian (second spatial derivative of the potential distribution[11], [47]), choosing the maximum degree of the Legendre polynomials to be 50, and the order of splines to be 4. To assess the presence of deviance-related effects on SCDs in the MMN and P3a time-windows, two-tailed *t*-tests were performed.

Aiming to reveal the generators of MMN and P3a, we applied brain electrical tomography (BET) analyses by means of the VARETA approach[12]–[14]. With this technique, sources are reconstructed by finding a discrete spline-interpolated solution to the EEG inverse problem: estimating the spatially smoothest intracranial primary current density (PCD) distribution compatible with the observed scalp voltages. This allows for point-to-point variation in the amount of spatial smoothness and restricts the allowable solutions to the grey matter (based on the probabilistic brain tissue maps available from the Montreal Neurological Institute[48]). This procedure minimizes the possibility of “ghost sources”, which are often present in linear inverse solutions[14]. A 3D grid of 3244 points (voxels, 7 mm grid spacing), representing possible sources of the scalp potential, and the recording array of 128 electrodes were registered with the average probabilistic brain atlas developed at the Montreal Neurological Institute. Subsequently, the scalp potentials for MMN and P3a were transformed into source space (at the predefined 3D grid locations) using VARETA. For both MMN and P3a, statistical parametric maps (SPMs) of the PCD estimates were constructed based on a voxel by voxel Hotelling *T^2^* test against zero in order to localize the sources of the component separately for each condition. For all SPMs, we used Random Field Theory[49] to correct activation threshold for spatial dependencies between voxels. We show results as 3D activation images constructed on the basis of the average brain.

## Supporting Information

Figure S1 Ignore condition for concrete rules. a) Grand-average ERPs elicited by standards (black) and deviants (gray), and deviant minus standard difference wave (red). b) Topographic distributions of the MMN and P3a components (scalp current density, SCD). Electrode positions with SCD values significantly deviating from zero are indicated in the p-value maps. c) Tomographic distributions of the MMN and P3a components (primary current density, PCD). The hotter colors correspond to higher probability values (one-way ANOVA; thresholded to p<.0001). Note the similarity of the MMN to that elicited by deviations from abstract rules. In contrast to the abstract rule sequences, P3a was elicited.
(1.38 MB TIF)

Figure S2 Distraction condition for concrete rules. a) Grand-average ERPs elicited by standards (black) and deviants (gray), and deviant minus standard difference wave (red). b) Topographic distributions of the MMN and P3a components (scalp current density, SCD). Electrode positions with SCD values significantly deviating from zero are indicated in the p-value maps. c) Tomographic distributions of the MMN and P3a components (primary current density, PCD). The hotter colors correspond to higher probability values (one-way ANOVA; thresholded to p<.0001). d) Behavioral data for standard and deviant tone pairs. Note the similarity of the MMN to that elicited by deviations from abstract rules.
(1.46 MB TIF)

Figure S3 Detection condition for concrete rules. a) Grand-average ERPs elicited by standards (black) and deviants (gray), and deviant minus standard difference wave (red) for detected deviants. ERPs for undetected deviants are not shown because they occurred too rarely (98% correctly indicated deviants). b) Topographic distributions of the MMN and P3a components for detected deviants (scalp current density, SCD). Electrode positions with SCD values significantly deviating from zero are indicated in the p-value maps. c) Tomographic distributions of the MMN and P3a components for detected deviants (primary current density, PCD). The hotter colors correspond to higher probability values (one-way ANOVA; thresholded to p<.0001). Note the similarity of the MMN to that elicited by deviations from abstract rules.
(1.33 MB TIF)

Figure S4 Detection patterns. In the abstract detection condition, a higher proportion of deviant tone pairs was registered when the second tone in the pair was long (left panel), and when the absolute frequency difference to the preceding tone pair was large as determined by a median split per subject (right panel). Significance of the differences in detection performance was verified by two-tailed paired Student's t-tests.
(0.16 MB TIF)
